# Potential and limiting factors in the use of alternative fuels in the European maritime sector

**DOI:** 10.1016/j.jclepro.2021.125849

**Published:** 2021-04-01

**Authors:** M. Prussi, N. Scarlat, M. Acciaro, V. Kosmas

**Affiliations:** a European Commission, Joint Research Centre (JRC), Ispra, Italy; b Kühne Logistics University KLU, Hamburg, Germany

**Keywords:** Biofuels, Maritime, Shipping, GHG savings, Alternative fuels

## Abstract

The maritime sector is a key asset for the world economy, but its environmental impact represents a major concern. The sector is primarily supplied with Heavy Fuel Oil, which results in high pollutant emissions. The sector has set targets for deacrbonisation, and alternative fuels have been identified as a short-to medium-term option. The paper addresses the complexity related to the activities of the maritime industry, and discusses the possible contribution of alternative fuels. A sector segmentation is proposed to define the consumption of each sub-segment, so to compare it with the current alternative fuel availability at European level. The paper shows that costs and GHG savings are fundamental enablers for the uptake of alternative fuels, but other aspects are also crucial: technical maturity, safety regulation, expertise needed, etc. The demand for alternative fuels has to be supported by an existing, reliable infrastructure, and this is not yet the case for many solutions (i.e. electricity, hydrogen or methanol). Various options are already available for maritime sector, but the future mix of fuels used will depend on technology improvements, availability, costs and the real potential for GHG emissions reduction.

## Highlight

• The paper proposes a reasoned definition of European maritime sector.
• Sector segmentation is proposed.
• Potential contribution of alternative fuels is assessed against current consumption.
• An analysis of the current limiting factors for alternative fuel uptake is provided.
• The future mix will depend on potential of various fuels for GHG emissions reduction.

## Graphical abstract

**Figure:**
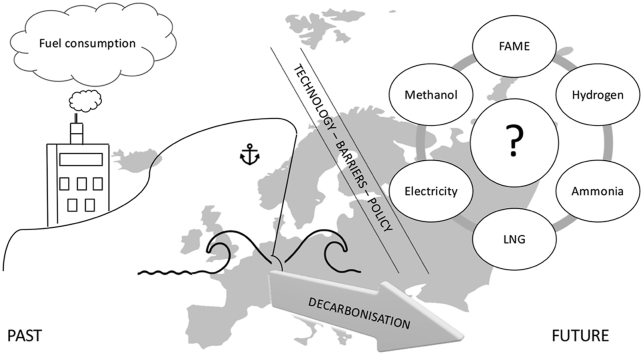

## 1 Introduction

The maritime sector is a key asset for the world economy. The definition of the maritime sector encompasses freight and passengers, and although the latter represents an important economic segment, the former is responsible for the largest part of emissions. According to Longva et al. (2014), the largest majority of international trade is seaborne, involving more than 85,000 registered vessels (Hsieh and Claus (2017)). At the European Union (EU) level, waterborne transport including domestic shipping and inland waterways, moves nearly 75% of external EU trade and 40% of internal EU trade (EC (2019c)). European maritime shipping companies control around 36% of the global fleet, and the EU maritime industry is estimated to contribute to more than 1% to the EU’s GDP, employing 2.1 million people (EC (2019b)).

Waterborne transport (including inland waterways) is generally consider energy efficient, when compared to road transport and aviation and when greenhouse gas (GHG) emissions (per tonne-km) are used as metric. In spite of the relative good efficiency of the propulsion systems, the use of Heavy Fuel Oil (HFO) (which is considered as a low quality grade fuel) resulted in high pollutant emissions (e.g., CO_2_, SOx, NOx), and consequently high environmental impacts (Toscano and Murena, 2019). Between 14 and 31% of the global emissions of NOx, and 4–9% of SOx, originate from marine vessels (Gilbert et al. (2018); Taljegård et al. (2015)). The industry consumes 330 Mt of marine fuel a year (Hsieh and Claus (2017)), of which the largest part (77%) is HFO. According to the same author, this energy demand is estimated to be responsible for 2–3% of global CO_2_ while other authors report even higher figures: 3–6% (Gilbert et al. (2018)). CO_2_ emissions from shipping are projected to rise in the range of 1.1–3.7 Gt CO_2_ /yr in 2050, with a 270% increase compared to 2007, in the business as usual scenario (Rehmatulla and Smith (2015)).

In order to tackle the severe effects of GHG on climate, the Paris Agreement (Unfccc (2019)) aimed to limit the increase in the global average temperature to well below 2 °C, above pre-industrial levels and to pursue efforts to limit the temperature increase to 1.5 °C. Waterborne transport is expected to contribute to the Paris Agreement targets, as well as to the achievement of the Sustainable Development Goals (SDG) of the United Nations Development Programme (UNDP) (United Nation (2020)).

The International Maritime Organisation (IMO)’s Marine Environment Protection Committee (MEPC) adopted in 2018 (IMO (2018)) an initial strategy, on the reduction of GHGs emissions from (seagoing) ships, setting out a vision for 2050. The strategy identifies three levels of ambition:

1. *“carbon intensity of the ship to decline by implementing energy efficiency design index (EEDI) […]”;*
2. *“carbon intensity of international shipping to decline to reduce CO*_*2*_ *[…] by at least 40% by 2030, pursuing efforts towards 70% by 2050 (compared to 2008)”*.
3. *“GHG emissions from international shipping to peak and decline as soon as possible, and to reduce their total annual by at least 50% by 2050 (compared to 2008) whilst pursuing efforts towards phasing them out […]”*.

The implementation of the emission control areas (ECA), established in 2005 was another important pillar of the sector’s strategy to reduce environmental impacts. This area was implemented in order to reduce emission of Sulphur oxides (SOx), Nitrogen oxides (NOx), and particulate matter (PM). In October 2016, IMO MEPC adopted the decision to reduce as of 1 January 2020 the Sulphur content of marine fuels down to 0.50% as in Europe (IMO (2020)). This resolution is expected to have a significant impact on fuels used by ships.

While the IMO has been addressing the issue, pursuing an international agreement, at European level the possible contribution of the maritime sector to the decarbonisation goals is under definition. In the European Commission communication *“Clear strategic long-term vision for a prosperous, modern, competitive and climate-neutral economy” A Clean Planet for All* (EC (2019a)), the marine sector is called to contribute to transport decarbonisation. Based on the scenarios drawn in this communication, in 2019 the new European Commission launched the “European Green Deal” (EC (2019d)), with the goal to ensure that Europe will be climate neutral by 2050. Additionally, the Renewable Energy Directive recast (2018/2001/EU) sets, for the first time, a specific multiplier (1.2X) to stimulate the EU maritime sector to contribute towards a 14% renewable energy penetration into the EU transport. The multiplier means that 1 MJ of biofuel used in the maritime sector can account 1.2 times for the reduction targets set by a Member State; this is supposed to foster alternative fuel uptake in the sector.

In spite of the ambitious targets set by industry and institutions, real alternative fuels uptake is today almost negligible on a commercial scale. Many factors influence alternative fuels market penetration, but existing literature mainly focus on costs and GHG saving potential only. The paper complements the current body of knowledge by presenting other technical and non-technical aspects, also highlighting the potential interactions with other transport modes (e.g. road and aviation).

Among the alternative fuels proposed, only few can today rely on a large scale production capacity, so comparing the broad volumes required by a certain segment could give a realistic picture of the potential contribution for a specif solution. It is worth noticing that the maritime sector is usually described as homogeneous, but actually provides various services and the ships are significantly different, both in terms of engines types and fuel demand. This diversity is the reason why the paper estimates the European demand for various sector segments. In the paper, the consumption of each sub-segment is compared with the information currently available for alternative fuels. Additionally, in the scientific and technical literature, many alternative options are currently proposed but without a clear vision on real GHG saving potentials (e.g. LNG), and the paper aims to clarify on this aspect.

All in all, the paper addresses the complexity and high variance related to the activities of the maritime industry, defines current fuel consumption and investigates the potential of current European alternative fuels to contribute to the decarbonisation of the maritime sector.

## 2 Alternative fuels for sector decarbonisation

**Fig. 1:**
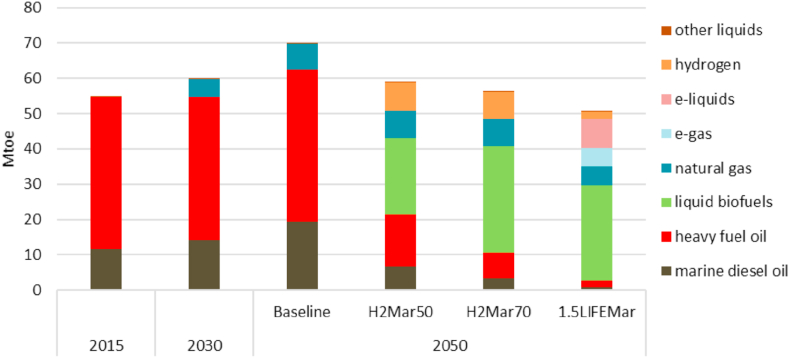
EU international maritime fuel mix in the Baseline and decarbonisation variants (source: EC (2019a)).

With a few exceptions, the shipping sector currently relies on internal combustion engines (ICE), supplied with petroleum-derived fuels. If this propulsion technology continues to dominate the sector, biofuels, e-methanol or ammonia could be used as a tool towards decarbonisation. IMO ambitions are based on high expectations for technological innovation in the sector, and on the global introduction of alternative fuels for international shipping (Faber et al. (2019)). According to the same source, liquid fuels are expected to be the main source of energy in the global maritime transport, as well as intra-EU and inland shipping. The more ambitious the CO_2_ reduction goal for 2050 (−80% GHG emissions or net zero (GHG emissions)), the higher the share of low-carbon fuels. Together with energy efficiency improvements, operational and technical optimisation (hull design, vessel size, engines and routing optimisationa), alternative fuels can play a crucial role in decarbonising the shipping sector. The European Commission 2050 long-term strategy baseline scenario reports a significant expected contribution of liquid alternative fuels for the sector (EC (2019a)). In this scenario proposed by the EC, three variants are proposed: H2Mar50, H2Mar70 and 1.5LIFEMar, based on high uptake of Hydrogen $H_{2}$ and on the 1.5 degree of global temperature increment LIFE scenario. In all the three variants, a significant uptake of liquid biofuels in the fuel mix by 2050 is expected: 37% of the energy demand in H2Mar50 and 54% in H2Mar70 and 1.5LIFEMa (Fig. 1).

In comparison with other transport modes (e.g. aviation), from a mere technical point of view, shipping is more flexible in terms of fuel supply. Today there are different alternative fuel options already available for shipping, including: biomethanol, ammonia, dimethyl ether (DME), biodiesel, and gaseous fuels such as LNG (liquefied natural gas) and bio-LNG, among others. Electricity can also be considered a suitable energy vector, mainly for short-haul regional trades. The altearntive fuels considered in this study are presented in Table 1.

**Table 1:** Alternative fuels for the shipping sector.

Type of Fuel	Source	Comments
HFO	Fossil sources	High impacting fuel
LNG	Fossil sources	Interesting gaseous alternative fuel
	Bio-derived	
Methanol	Fossil sources	Low density liquid alternative fuel
	Bio-derived	
FAME	(Biodiesel)	Widely used road alternate fuel
HVO	Hydrotreated Vegetable Oil	Drop-in fuel widely used in road and aviation
Ammonia	Synthesis	New potential alternative fuel for ships
Electricity	Various source	Energy vector
Hydrogen	from Natural Gas	Energy vector
	from renewables	

It is worth mentioning that, according to EC (2019b), while innovative technologies and alternative fuels are already available, a full sector climate neutrality by 2050 would be a challenging target, considering the average lifetime of a modern ship is 25–30 years, and the growth rate of the sector. Additionally, such target requires that not only vessels will have to be ready for implementing the changes (i.e. through fleet renewal, or retrofitting), but also ports, terminals, etc.

## 3 Materials and methods

Categorizing maritime related activities is a complex task, this work is based on data and information derived from various literature sources (studies, reports, etc.) and policy documents, on the environmental performance of the maritime industry. A reasoned fleet segmentation, and the fuel used by the various types of ships, is proposed to define the European dimension of shipping. The review demonstrates that there is no consensus regarding the taxonomy of maritime related activities. Literature sources have been used to populate the JRC FF20 tool (described in the next section), in order to calculate fuel consumption for the proposed sector segments. Results for fuel consumption have been then put in relation with available figures for current alternative fuels.

### 3.1 Definition of maritime EU dimension

The European maritime sector is usually defined as domestic shipping and inland waterways. For the purpose of this paper, however, we refer to a more detailed definition, proposed in one of the latest policies that have come into force at the EU level, which is the Monitoring, Reporting and Verification (MRV) of CO_2_ emissions (Regulation (EU) 2015/757 of the European Parliament and the Council of 29 April 2015 on the monitoring, reporting and verification of carbon dioxide emissions from maritime transport, and amending Directive 2009/16/EC). The regulation applies to “*All intra-Union voyages, all incoming voyages from the last non-Union port to the first Union port of call and all outgoing voyages from a Union port to the next non-Union port of call, including ballast voyages*” (EC, 2020). It applies to both cargo and passenger vessels over 5000 gross tonnage (GT), regardless of their flag, but not to ships utilised for dredging, ice-breaking, pipe laying or offshore installation activities, warships, naval auxiliaries, fish-catching or fish-processing, wooden ships of a primitive build, ships not propelled by mechanical means, or government ships used for non-commercial purposes.

Table 2 reports considerations based on previous and additional sources (e.g. EU (1999)). Additionally, due to the importance of the fishing sector (Romeu et al., 2019) and the possibility to include this in the MRV regulation, in spite of the information scarcity, the paper presents an estimation of its impact. All this considered, the option nr. 4 allows to get a wider picture of the EU shipping sector, and it has therefore been preferred.

**Table 2:** Different proposals for a definition of the EU maritime sector.

**Options**				
1	2	3	4	5
All intra-EU voyages (as described in the MRV)				
Ships only above	All ships (that can be traced), regardless the flag			
5000 GT, regardless				
the flag.				
No Fishing		Fishing		No Fishing
No inland waterways			Inland waterways	

### 3.2 The FF20 maritime model

The JRC has been developing its tool for “Fleets and Fuels” (FF20) modeling, to estimate final energy consumption (Mtoe) for a specific segment of the transport sectors. FF20 is a modeling tool using linear equations, which starts from the definition of the fleet for a certain transport mode, and associates efficiency and activities to each sub-segment, to determine the energy consumption and creating scenarios for alternative fuels uptake. This tool is suitable for creating scenarios for alternative fuels uptake in various sectors, namely road, maritime and aviation. The fundamental equation used for defining energy consumption is:

(1) EC=Ac⋅St⋅SC

where:

• Ac:activity[tkmpership]
• St:stock[shipsnr]
• SC:SpecificfuelConsumption[MJ/tkm]

In the model, the fleet stock (expressed as the number of vessels at a certain age) for a given year is a pre-set variable, defined by the data available from the literature. To define the stock of a specific segment, for a specific year, the information needed is:

• Overall stock for the reference year (e.g. 2019): nr. of ships;
• Average age of the fleet, in order to tune the so called “scrappage function”.

The Specific Consumption (SC) is a pre-set fixed variable. This can be defined per segment and/or per type of engine and/or per type of ship. Activity is another input, representing the total activity for a certain segment. The model distributes the total stock along a certain time interval (usually 30 years), as to create a stock representing fleet dimension and performances (average age of the vessel represented through the age). A scrappage function can be applied to remove older ships from the stock and introduce new ships. This will allow to change the technologies applied to the stock, over time, and to better take the reported average age into consideration. Fine tuning and verification have been carried out by adjusting inputs, to align the resulting final Energy Consumption (EC) with other studies (e.g. POTEnCIA model JRC (2019)).

### 3.3 Fleet definition and fuel demand

According to International Energy Agency (Hsieh and Claus (2017)), until a few decades ago, ships were commonly used as a transporter of people, while nowadays the sector is largely devoted to move freights. Because of this trend, and technological improvements, the average size of ships has also increased substantially over the past decades: larger vessels reduce shipping costs per load unit, as well as operational and maintenance costs (Hsieh and Claus (2017)), as long as there is an adequate utilisation of ship capacity. Vessels specialisation also increased, for instance today refrigerated cargo ships, roll-on/roll-off (ro-ro), gas carriers designed to transport liquefied bulk chemical gases (e.g LNG and LPG), etc. are available.

The aim of this section is to propose a fleet model, with a segmentation into several sub-segments. THETIS-MRV (EC (2020)) and other databases have been elaborated in order to define a total fleet, in terms of the total number of ships moving across the identified area of interest for this report. In order to characterise the fleet type, ships have been grouped together and seven categories have been defined. For fishing (Romeu et al. (2019)) has been used as main source of information. The total fuel consumed by the EU fishing fleet was about 2 million tonnes of almost entirely marine gas oil (diesel) (Romeu et al. (2019)).

Based on elaboration of the existing literature (EC (2020); Romeu et al. (2019)), the follow segmentation is proposed in Table 3.

**Table 3:** Fleet composition.

Segment	Type of Ship	Nr. Of ships
B	Bulkers	3675
C	Cargo/Container	3871
T	Tankers	3615
R	Ro-ro/Ro-Pax	339
F	Fishing	65,567
P	Passengers	152
IWW	Inland Water Ways	–

FF20 has been used to determine the fuel consumption of each segment. Input data on activity are required to calculate the final consumption, given a certain average fleet efficiency; central scenario of POTEnCIA (JRC (2019)) segments maritime sector in the EU has been used to derive such information. In order to validate assumptions about activity and efficiencies, the resulting fuel consumption has been compared with MRV values, which are in line with the model estimates. Data for Inland Water Ways (IWW) have been derived from POTEnCIA (JRC (2019)). Information about various class of ships, as well as their efficiencies, has been extracted from THETIS-MRV report (EC (2020)). To derive a representative average efficiency for each class of ship, the resulting consumption from THETIS-MRV have been compared with the POTEnCIA model results.

Based on the above described inputs, the values used in the model for calculating consumption are reported in Table 4. For fishing and inland waterways, mainly due to the lack of information, the consumption have been reported as aggregated value.

**Table 4:** Inputs for estimating fleet fuel consumption.

Segment	Type of Ship	Total Distance
*-*	*-*	*Mtkm*
B	Bulkers	1,995,069
C	General Cargo/Container	7,348,897
T	Tankers	3,945,940
R	Ro-ro/Ro-Pax	1,523,610
F	Fishing	–
P	Passengers	710,659
IWW	Inland Water Ways	156,767

Fuel type is another important piece of information needed to draw alternative fuels uptake scenarios (Işıklı et al. (2020)). Almost 70% of the fuel consumed in Europe and reported in THETIS-MRV (EC (2020)) is HFO, while the rest is gas oil and liquefied natural gas.

Based on the information reported in THETIS-MRV (and the emission factors used), it has been possible to derive the average percentage of HFO, gas oil and LNG used by each class of ships. The results from simulations are reported in Table 5. From the elaboration reported in the table, it is clear that segment C dominates the sector consumption, in spite of a limited number of vessels. This is mostly related to the high activity associated with this segment. After general cargo/containers (44%), T (26.3%), B (12%), segments R, F, P and IWW together represent about 20% of consumption. It is worth highlighting that, in line with Romeu et al. (2019), fuel consumption is about 2 million tonnes of almost entirely gas oil (diesel).

**Table 5:** Final fuel consumption per class (ktoe).

Segment	Type of Ship	Total Fuel	%	HFO	Gas oil	LNG
B	**Bulkers**	5746	12.0	4597	1149	0
C	**Cargo/Container**	21,166	44.0	15,963	4708	495
T	**Tankers**	11,365	26.3	6887	2844	1634
R	**Ro-ro/Ro-Pax**	4388	9.1	3072	1317	1
F	**Fishing**	2196	4.6	220	1976	0
P	**Passengers**	2047	4.3	1433	614	0
IWW	**Inland Water Ways**	1156	2.4	215	1935	0
–	**Total**	48,064	–	32,387	14,543	2130

## 4 Alternative fuel uptake

Alternative fuels for maritime transport encompass any fuel suitable for the provision of existing services, potentially offering environmental benefits when compared against business as usual scenario; the alternative fuels considered in this study have been presented in Table 1. There are multiple factors influencing the market uptake of alternative fuels, some of which are specific for the maritime sector while others are common to road and aviation.

**Fig. 2:**
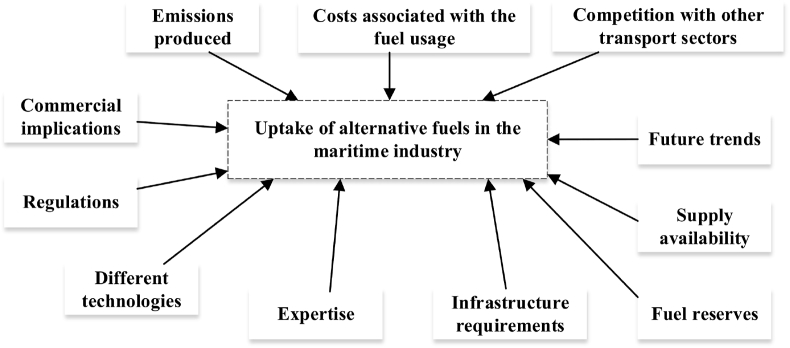
Framework of the uptake of alternative fuels (source: KLU report for JRC).

The vast majority of the available literature focus on the cost differential for the alternative fuels against the HFO and diesel, and the potential environmental benefits of the proposed solutions. Costs are broadly accepted as the main enabling factors for fuel market penetration but there are several others factors that need to be taken into consideration, among others: specific engine requirements, regulatory drivers, fuel supply availability, volume requirements according to ship size and industry’s expertise, etc. The approach used in the current analysis is presented in Fig. 2.

According to this approach, the following issues have been considered, in the comparative analysis of alternative fuels presented in Table 6.

• Emissions: well-to-tank (WTT) and tank-to-propeller (TTP) should be evaluated against the regulatory framework.
• Costs: the important determinants of costs are linked to current price differentials with oil-based fuels and the expected cost increase associated with regulation.
• Availability in relation to use in other sectors (e.g. road and aviation): the issue of interdependent demand may be critical for shipping as some of the fuel alternatives could be used by other sectors, hence reducing the availability of the fuel.
• Supply availability of the fuel.
• Port infrastructure and refueling points.
• Expertise: fuel knowledge will be an important factor affecting its uptake, both in terms of onboard handling as well as among ship owners and operators.
• Technical maturity of the fuel for maritime use: ships have specific technical characteristics that impose constraints on the use of alternative fuels. These relate to safety, handling of low flashpoint fuels, use of space on board of the ship, lost capacity, autonomy, etc.
• Future fuel market trends: fuel blending is also critical for the uptake of alternative fuels in shipping.
• Regulation: expected to contribute to shape some of the framework conditions in which the sector will operate and develop.
• Competition with other low-carbon technologies: The uptake of alternative fuels depends on the rapidity with which other low-carbon technologies will be deployed (e.g. wind propulsion, batteries).

**Table 6:**
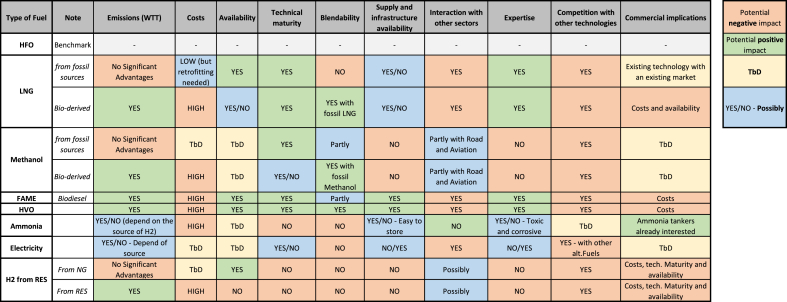
Comparative analysis of alternative fuels for shipping sector.

Table 6 reports the summary of the analysis, and it aims highlighting the potential positive and negative impacts of several alternative fuels, in relation to the described aspects.

The emission saving potential of the alternative fuels have not been defined yet, at IMO level. Greenhouse gas reduction potential for these fuels have been investigated mainly for road applications (Prussi et al. (2020); Argonne National Laboratory (2020)), while at the moment only a few studies have looked into the maritime sector (e.g. Bouman et al. (2017); Nair and Acciaro (2018)).

Liquefied natural gas (LNG) is one of the available alternative fuels already used in ships, with several clear advantages with respect to other options. However when it is produced from fossil sources its GHG saving potential is almost negligible (Brynolf et al. (2014)). Conversely, the use of liquefied biomethane has the possibility to offer relevant saving (Prussi et al. (2020)), but its availability is currently limited. As LNG requires relevant on-board modification, it is more suitable for certain subsegments (e.g. containers, tankers). The consumption estimated for these segments, as presented in Table 5, currently does not fit with the estimated availability of biomethane at European level (Prussi et al. (2019)). LNG can be considered as a transitional fuel towards a full decarbonisation, while paving the way for bio-derived LNG. Nevertheless, it is worth remarking that the undesired releases of methane during the operational phases can significantly reduce any potential advantage.

Apart from GHG emissions, combustion engines emits also other local pollutants (i.e PMs Viana et al. (2020)) and alternative fuels can only contribute to reduce these to a small amount. It worth remarking that alternative fuels are one of the technical option to limit emissions, Hongrui et al. (2012) showed that a scrubber system, used with current heavy fuel oils, has a significant potential to reduce emissions with low well-to-wake energy consumption. In order to reduce global (GHGs) but also local pollutant, solution such as electricity and hydrogen fuel cells are usually proposed as zero emission options (JRC (2020)). Faber et al. (2019) reports three ways for producing zero emission fuels:

• using renewable electricity either directly or in a electrochemical process to generate fuel (e-fuels);
• plants converting solar energy into a biological fuel precursor (e.g. algae);
• producing hydrogen by reforming methane or other hydrocarbons and store the CO_2_ (CCS).

It has to be noted that the use of the term “zero emissions” is strictly correct only from a Tank-to-Wake perspective, as the upstream emissions (Well-to-Tank) may occur. In general, electricity and hydrogen should be primarily considered as energy carriers, with environmental performances determined by the primary source used for their production but upstream emissions for the production of thee energy vectors. Their environmental performances are determined by the primary source used for their production so do not lead to any advantages, if the primary energy is not from a carbon-neutral source. Similarly, and from a mere GHG reduction perspective, the use of hydrogen fuel cells may not lead to any advantages, if the electricity used for its production is not generated from a carbon-neutral source.

Fuel costs, as already highlighted, represent a major expense for shipping (Notteboom and Vernimmen (2009)), and today the most important limiting factor for alternative fuels to be competitive. The considerations reported in Table 6 are based on several studies, among others Helgason et al. (2020), reporting the cost differential between fossil and fuels derived from renewable natural gas, and the evaluation about total cost of ownership from JRC (2020) and IEA (2020), and the recent sensitivity analyses proposed by Trapp et al. (2020).

Real availability of alternative fuels for shipping, in terms of scale, is not clear today for most of the pathways, as demand has not been defined yet. It is necessary to highlight that current biofuels consumption in Europe accounts for 15.4 Mtoe (in 2017 (EurObservER, 2018)), so any additional demand from the maritime sector, according to estimated consumption is expected to impact the whole alternative fuel sector. Additionally, it is worth noting that strong competition is expected to occur in existing markets, in particular for alternative fuels currently used in road or aviation (Hsieh and Claus (2017) and Bengtsson et al. (2014)).

Based on described literature and experts judgments, Table 6 reports also considerations on technical maturity and available infrastructure, as these are other relevant factors, as highlighted in several studies (e.g. Chryssakis et al. (2014)), and on the possible lack of infrastructure and the increased complexity in managing alternative fuels onboard of the vessels (the latter mainly in relation to safety issues associated with the use of such fuels). These are aspects relevant for ammonia and hydrogen in particular. Liquids options such as FAME are constrained by technical blending limits (mainly related to storability in marine environment), while HVO as a drop-in fuel could be used in higher concentration. Alcohols (e.g. methanol and ethanol) and derived ethers could also be considered, preferably if obtained from renewable sources. According to several studies (i.e. JEC WTT study Prussi et al. (2020)), the Technological and Commercial Readiness Levels (TRL and CRL) for electricity, hydrogen and e-fuel pathways are far from being close to market. Pilot initiatives are already under advance development, such as the Horizon 2020-funded ‘E-ferry’ project (H2020 project (2019)). The e-ferry project was about the design, building and demonstration of a fully electric powered ferry for 200 passengers, which entered in operation in 2019. However, deploying zero-emission vessels is today easier for short-sea journeys (EC (2019b)) than for freight segment. However, considering the need of developing a port infrastructure and distribution onland full electrification remains challenging today. Additionally, as for the road freight sector, deep-sea journeys require much higher-density power sources and current technical obstacles may limit the development of this alternative.

All in all, it is clear that the current status of knowledge does not allow providing a clear scenario, as practically all existing alternatives share many constraints, limiting their competitiveness against other technologies and existing fuels.

## 5 Conclusions

Environmental impacts of maritime sector represents today a major concern at both EU and global level. The IMO and the European Commission are acting to stimulate and support the sector in the transition towards significant greenhouse gas emissions reduction. In order to meet these targets, the shipping sector has to shift from relying on fossil fuels to using alternative fuels, as internal combustion engines are expected to remain relevant in the medium term. Today, in spite of these targets, the uptake of alternative fuels is not significant at commercial scale. Real penetration of a specific alternative fuel will be defined by an array of technical, and non-technical factors.

The paper illustrates that even if cost and GHG saving are fundamental enablers to the fuel uptake, other aspects such as technical maturity, safety regulations, operators expertise, etc. are not sufficiently analysed for certain solutions (e.g. ammonia, hydrogen). Additionally, estimation of current segment demand has to be compared with current production capacity, to obtain a realistic picture of the potential contribution for a specif alternative fuel (e.g. hydrogen, bio-LNG). A demand for alternative fuel has to be supported by an existing reliable infrastructure, and this is not ready yet for most solutions (e.g. electricity or methanol).

While various options are already available for maritime transport, the future mix of fuels used in transport will depend on technology improvements, availability, costs and the potential of various fuels for GHG emission reduction.

The present article paves also the way for future research regarding alternative fuels usage within the maritime sector. Particularly, future studies could focus on the validation and expansion of the comparative analysis provided in this paper. Last but not least, a quantitative modelling approach could be applied for the determination of the potential future fuels mix in the industry.

## Disclaimer

The views expressed here are purely those of the authors and may not, under any circumstances, be regarded as an official position of the European Commission.

## Credit author statement

Matteo Prussi (corresponding author): Conceptualization, Methodology, Writing- Original draft and final preparation. Michele Acciaro Conceptualization, Data curation, Writing. Vasileios Kosmas: Writing - Review & Editing. Nicolae Scarlat Review & Editing, Supervision.

## Declaration of competing interest

The authors declare that they have no known competing financial interests or personal relationships that could have appeared to influence the work reported in this paper.

## Figures and Tables

**Table 7:** Comparison of the maritime sector definitions.

**Sources**		
Study commissioned by the European Commission-DG Mare (Policy Research Corporation, 2008)	Project by the European Network of Maritime Clusters- Netherlands Maritime Technology (Blonk, 2015)	Academic Textbook (Stopford, 2009)
**Maritime related activities**		
		**Vessel operations**
*Shipping*	*Seagoing shipping*	*Merchant shipping*
		*Cruise*
*Seaports*	*Sea ports*	*Ports*
*Navy*	*Navy*	*Navy*
		**Shipbuilding**
*Shipbuilding*	*Shipbuilding*	*Merchant and naval shipbuilding*
*Marine equipment*	*Maritime supplier industries*	*Marine equipment*
		**Marine resource**
*Offshore supply*	*Offshore*	*Offshore oil, gas*
		*Renewable energy*
*Marine aggregates*		*Minerals and aggregates*
		**Marine fisheries**
	*Fisheries*	*Fishing*
		*Aquaculture, seafood processing, etc*.
		**Other**
*Recreational boating*	*Watersports industry*	*Tourism, marine services, etc.*
*Marine services*	*Maritime services*	
*Inland navigation*	*Inland shipping*	
*Maritime works*	*Dredging industry*	

## Appendix Sector segmentation

Table 7 illustrates the differences found in the various approaches, from official documents and literature.
